# Supplementary material for: Sources of bias in artificial intelligence that perpetuate healthcare disparities—A global review
Source: PLOS Digit Health. 2022 Mar 31;1(3):e0000022. doi: 10.1371/journal.pdig.0000022 (PMC9931338; doi:10.1371/journal.pdig.0000022)
Supplement: Attachment — Submitted filename: 10nov21_ML_reply.docx [file pdig.0000022.s001.docx]

| EDITORIAL TEAM COMMENTS (note: line references refer to the *tracked* manuscript) | | |
| --- | --- | --- |
| Comment | Response | Line |
| Please amend your detailed Financial Disclosure statement. This is published with the article, therefore should be completed in full sentences and contain the exact wording you wish to be published. | Please see amendment on line 903 (also see below). | 903 |
| Please include all sources of funding (financial or material support) for your study. List the grants (with grant number) or organizations (with url) that supported your study, including funding received from your institution.  ii). State the initials, alongside each funding source, of each author to receive each grant. iii). State what role the funders took in the study. If no role in your study, please state: “The funders had no role in study design, data collection and analysis, decision to publish, or preparation of the manuscript.” iv). If any authors received a salary from funders, please state which authors and which funders.  If you did not receive any funding for this study, please simply state: “The authors received no specific funding for this work.” | Please see amendment:  “*Financial Disclosures*  LAC is funded by NIBIB grant R01 EB017205. The funders of the grant had no role in study design, data collection and analysis, decision to publish, or preparation of the manuscript. Grant detail can be found at: https://grantome.com/grant/NIH/R01-EB017205-01A1  No other authors received any specific funding for this work”. | 903 |
| Please update the completed 'Competing Interests' statement, including any COIs declared by your co-authors. If you have no competing interests to declare, please state "The authors have declared that no competing interests exist". Otherwise please declare all competing interests beginning with the statement "I have read the journal's policy and the authors of this manuscript have the following competing interests:" | Please see line 899. No competing interests exist.  “The authors have declared that no competing interests exist”. | 899 |
| Please provide separate figure files in .tif or .eps format only and remove any figures embedded in your manuscript file.  Please ensure that all files are under our size limit of 20MB.  Once you've converted your files to .tif or .eps, please also make sure that your figures meet our format requirements | Please find all figures uploaded as separate .tif files. | -- |
| Tables should not be uploaded as individual files. Please remove these files and include the tables in your manuscript file. | Please find table attached in the manuscript file (from line 912) | 912 |
| We have noticed that you have cited 'Supplementary Figure X' in the manuscript file. However, there is no corresponding file uploaded to the submission. Please ensure that all files are present to ensure that your paper is fully reviewed. | We apologise – this was from a much older draft and was overlooked in the final version. The mention of the supplementary figure has been removed. | 276 |
| Please review your reference list to ensure that it is complete and correct. If you have cited papers that have been retracted, please include the rationale for doing so in the manuscript text, or remove these references and replace them with relevant current references. Any changes to the reference list should be mentioned in the rebuttal letter that accompanies your revised manuscript. If you need to cite a retracted article, indicate the article’s retracted status in the References list and also include a citation and full reference for the retraction notice. | All references are correctly listed. Thank you for outlining this to us. | -- |
| This is a timely piece and with the addition of text descriptions and clarifications, and perhaps a slight change in the title, will be a great contribution to the literature. In particular additional reflections on the potential gaps in the available methods for detecting bias in medical ML studies such as nationality of authors vs country of current institutional affiliation, and the accuracy of gender based on first names from multiple ethnicities. | We thank the editorial team for their support of our article. Please find below our point-by-point response to reviewer comments, which we all feel improve the quality of the manuscript.  We hope we have addressed each of the reviewer points suitably. | -- |

| REVIEWER 1 (note: line references refer to the *tracked* manuscript) | | |
| --- | --- | --- |
| Comment | Response | Line |
| The sources of bias in AI research methodology that were highlighted in the body of this paper (but not clearly stated in the title) are found in the beginning of any AI clinical study. This is the part of the methodology where humans are involved—in the conception of the AI clinical study design and in the generation of the hypothesis (H1) to be tested. Another source of bias is the creation of the AI model that fails to take into account inherent differences in disease characteristics in human populations from which training, internal validation and testing data is gathered vs. the disease characteristics in human populations where the study findings will be applied. | We thank the reviewer for highlighting the over-arching message of our manuscript, noting that sources of bias in AI/ML in healthcare come from both (i) pointe where humans are involved in data collection and inclusion in model building, and (ii) systematic biases in the data being fed to the models – it being relatively homogeneous, and poorly-representative of patient/disease-differences between overrepresented and underrepresented patient populations. We agree that this emphasises the need to develop infrastructure for data-poor regions to collect and contribute data toward such models, and for model validation and re-calibration in the meanwhile. | -- |
| What may be a more suitable title, based on the methodology and findings of this study, is SOURCES OF BIAS IN ARTIFICIAL INTELLIGENCE CLINICAL STUDIES THAT PERPETUATE DISPARITIES IN HEALTHCARE. This study’s authors could then go on to identify 2 sources of bias in AI-powered studies: 1) the researchers who ask the clinical question and gather and analyze relevant data (authors’ clinical specialty, nationality, sex, expertise may introduce bias), and 2) the population of interest from whom the clinical data is gathered, which may have different disease characteristics from the general disease population upon whom the study findings will be applied (country source). A more thorough review of literature on the existence of disparities in global healthcare would serve as a relevant background to this study. | We thank the reviewer for their suggested title revision, which we believe more accurately describes the core message of our paper. We have changed out title accordingly:  “Sources of Bias in Artificial Intelligence that Perpetuate Healthcare Disparities – a Global Review”.  We also agree that summarizing the different sources of bias we have investigated as either (i) author-related, or (ii) population-related is a clear way to communicate causes of bias to our readers. We’ve outlined this suggested terminology in the opening line of our conclusion:  “Here, we demonstrate both population- and author-sources contributing toward bias in AI in healthcare. We find that U.S. and Chinese datasets and authors….”  Rather than explore current healthcare disparities at a global level in this manuscript, the authors wanted to maintain focus on the role for AI/ML in ameliorating those disparities (and risks of widening them, if used inappropriately). We have included 2 World Health Organization references in our introduction (including the World Health Statistics *Monitoring Health for the SDGs Report 2021)* – see: https://apps.who.int/iris/bitstream/handle/  10665/342703/9789240027053-eng.pdf | 1,  90,  693 |
| Regarding limitations of the study. This study uses AI to study the biases inherent in AI clinical studies. This begs the question: do the same biases apply to non-clinical AI studies such as this? Are the researchers of this non-clinical AI study biased because their study was conducted in the United States, because of the nationality (needs to be better defined) of the first and last authors, because of the gender of the first and last authors and because of their expertise? An introspective answer to these questions may further help in proving the point of this study, that all humans are biased, and that these biases affect the direction of healthcare research and application in the real world. | We appreciate the limitations to our study – especially to author country of origin, and that the first-listed authors’ affiliation does not necessarily reflect their country of origin (but rather, the institution they’re affiliated with at the time of publication).  Indeed, of our own author list, our authors were actually from Australia, Canada, Philippines, France, the US and Germany – but our *entire author list* describes our US-affiliations at the time of the manuscript composition and submission.  We have outlined with greater clarity the limitations to our own study, and the fact that these limitations to detail embedded into data (and subsequently, into AI) are often extremely difficult to account for, prior model development:  “Moreover, classification by clinical specialty may be restrictive and not holistically capture the many fields in which model-derived findings can apply. For example, an ML-based study of chest radiographs would augment a radiologist’s work but might also impact the care provided by a cardiologist, a pulmonologist, an internal medicine physician, and many other clinicians to their patients. Importantly, the gender inference methods used in this paper, as with any methods outside of direct survey and self-identification, are imperfect and likely to have misgendered a subset of the author population. Additionally, the Genderize.io API was unable to assign a gender to every author name. Further, the API is not inclusive beyond the gender binary (e.g., intersectional gender and they/them pronouns are not considered). We appreciate that author affiliation is not necessarily representative of author nationality, as it was used here to describe. Indeed, despite our own authors hailing from Australia, Canada, the U.S., Philippines, France and Germany – all of our affiliations suggest we are from the U.S. – highlighting further intrinsic biases in data, subsequently embedded into AI if not carefully accounted for”.  Thank you for raising this important point. | 670 |

| REVIEWER 2 (note: line references refer to the *tracked* manuscript) | | |
| --- | --- | --- |
| Comment | Response | Line |
| Thank you for the opportunity to review your paper on this important topic. The work behind this is considerable.  However, I think it needs a re-think in terms of context. The paper presented extols a single view, and I think needs a more balanced view - in effect the paper risks losing impact because it reads as biased in itself.  Much of the current general research literature is arguably biased or potentially biased - including populations, settings, authors, and institutions. Therefore, AI based research needs to be viewed through this lens. It is because of this bias that one of the key tenants of Evidence Based Medicine is the question ‘does this apply to my patient?’. AI research should be viewed through the same lens. By referring back to EBM, it can put AI based research into a similar context to other research, making it potentially more digestible and more mainstream. | We agree with the reviewer that our paper emphasises bias within data used for AI in healthcare, that it (in itself) could be perceived as one-sided. A balanced view contextualising how principles of EBM can be applied to AI in healthcare (“how do *these* findings apply to *my* patient?”) are important.  We have outlined the importance of understanding *when* to apply findings from models (built with homogeneous data) – per EBM principles (“how do these findings *apply to* my patient?”); line 322:  “We stress that while such disproportionate overrepresentation of U.S. and Chinese patient data in AI brings the risk of bias and disparity, it does *not* negate/undercut/undermine the value of understanding when and why these models remain beneficial, particularly in data-poor and demographically diverse regions (both within these nations and globally)”.  Further, we have discussed problems faced by physicians in understanding when to apply such ML findings to their patients, knowing the models have been built with relatively homogeneous data; line 522:  “If the physician clinically applying model predictions did not fully appreciate this risk, the model might hold undue (because unsubstantiated) influence over the decision to escalate or withdraw care. Alternatively, if this limitation *is* known, the model may simply not be used at all, thereby restricting benefits to the U.S. population upon whose data it was exclusively trained. Either outcome is disadvantageous to populations not represented in the large datasets commonly used to build these models”.  Please see our response to your fourth point (regarding transfer learning and customizing findings to diverse populations) re. how we have further expanded discussion of the importance of this.  However, we have outlined how unfortunately, appreciation of this risk appears to have been minimal, to date. Indeed, *half* of the datasets used to train COVID-19 outcome prediction models reported *no* sociodemographic features within their datasets, and *none of the studies* performed *any* adequate risk-of-bias assessment. The majority of those building models for AI in healthcare do not seem to be paying due (or any) regard to the importance of the question “how do these findings apply to my patient?”.  We have now outlined the benefits we stand to gain from the rapid evolution of AI in healthcare (especially those in resource-poor settings, with less specialist availability) – but through the lens of the importance of understanding when and how findings can be applied. Please find tracked changes (699), as below:  “The rapid evolution of AI in healthcare brings unprecedented opportunities for accurate, efficient, and cost-effective diagnosis superseding the accuracy of clinical specialists, but with minimal human input – especially valuable in resource-poor settings where specialist input is relatively more scarce. Although medicine stands to benefit immensely from publicly available anonymised data informing AI-based models, pervasive disparities in global datasets should be addressed”.  Please find further balanced viewpoints, as outlined below.  Thank you again for reading our manuscript so carefully, and such thoughtful insights. | 322,  522,  699 |
| The choice of current institution as representing the world view of the authors is potentially misleading. Looking at the authors of this paper there is broad cultural diversity, not represented in the list of affiliations. Therefore, this tool is creating its own bias. This needs to be recognised and discussed. The same may apply to the name API.  There are some developing tools to detect bias in datasets – something that is not represented in any previous non-AI research. Although these are still not often used, in a paper on bias I think their existence should be recognized, and use encouraged. | We appreciate the limitations to our study – especially to author country of origin, and that the first-listed authors’ affiliation does not necessarily reflect their country of origin (but rather, the institution they’re affiliated with at the time of publication).  Indeed, of our own author list, our authors were actually from Australia, Canada, Philippines, France, the US and Germany – but our *entire author list* describes our US-affiliations at the time of the manuscript composition and submission.  We have outlined with greater clarity the limitations to our own study, and the fact that these limitations to detail embedded into data (and subsequently, into AI) are often extremely difficult to account for, prior model development:  “Moreover, classification by clinical specialty may be restrictive and not holistically capture the many fields in which model-derived findings can apply. For example, an ML-based study of chest radiographs would augment a radiologist’s work but might also impact the care provided by a cardiologist, a pulmonologist, an internal medicine physician, and many other clinicians to their patients. Importantly, the gender inference methods used in this paper, as with any methods outside of direct survey and self-identification, are imperfect and likely to have misgendered a subset of the author population. Additionally, the Genderize.io API was unable to assign a gender to every author name. Further, the API is not inclusive beyond the gender binary (e.g., intersectional gender and they/them pronouns are not considered). We appreciate that author affiliation is not necessarily representative of author nationality, as it was used here to describe. Indeed, despite our own authors hailing from Australia, Canada, the U.S., Philippines, France and Germany – all of our affiliations suggest we are from the U.S. – highlighting further intrinsic biases in data, subsequently embedded into AI if not carefully accounted for”.  Thank you for raising this important point. | 670 |
| The growing availability of datasets also needs a balanced view. Noting that there are real and potential biases in these, it is also important to recognize these have already had a valuable contribution to the field, and their ongoing development and use should be encouraged rather than discouraged, noting the limitations. Their availability becomes an attractant to time investment, which potentially improves the opportunity to democratize Healthcare. | We acknowledge that the perspectives in our paper emphasise the risk of ML in healthcare to do harm (due to bias), and negate inspection of the immense benefit to stand from safe use of ML in healthcare.  We have added the following passage into our Discussion, to outline the extent of growth of data and computational power, and how this expands the capacity for growth of ML in healthcare (and associated benefits):  “Increased computer power and massive dataset availability has driven the growth of ML in healthcare, especially over the last decade. The volume of data being generated cannot be understated - 108 bytes in the U.S. alone, with annual growth of 48% - as well as increasing uptake of digital electronic healthcare systems and proliferation of medical devices. The ability of machines to interpret and manipulate data relatively-independent of human supervision/input brings with it the immense potential for improvements in healthcare quality and accessibility – particularly in resource-poor settings where specialist opinion is not as readily available”.  Please also find our additional note of this in our Conclusion:  “The rapid evolution of AI in healthcare brings unprecedented opportunities for accurate, efficient, and cost-effective diagnosis superseding the accuracy of clinical specialists, but with minimal human input – especially valuable in resource-poor settings where specialist input is relatively more scarce”.  Please find tracked changes in our manuscript.  We hope we have been able to outline the benefits of ML in healthcare, while also emphasising the need for thoughtful application (to avoid perpetuating healthcare inequalities).  Thank you for outlining this to us. We believe the manuscript presents a more balanced view and hope we have addressed comments suitably. | 530,  699 |
| The ability to transfer learn and thus customize to local populations needs more attention. This technique allows the power of large datasets to help inform smaller datasets, meaning it is simpler to get a return on investment in lower resource environments, thereby encouraging development in this area. It also leverages the investment in resource rich environments. Also, of note is that for most non-AI interventions this opportunity rarely if ever exists – either to repeat the intervention, or tailor the intervention to local population and parameters. | We thank the reviewer for drawing attention to this, and agree that not only have model pre-training approaches and transfer learning helped bring substantial performance gains in clinical medicine prediction tasks, but also broadened access to healthcare machine learning and its applications in low-resource settings.  In addition to the wide utility of AI-based models in clinical medicine, the need to have readily available and high-quality pre-processed datasets and high training costs jointly motivate/prompt model structure/parameter sharing and open-access release.  We believe this is worth particular mention, and have thus incorporated such into our Discussion (see tracked changes, and below). We have also made multiple changes throughout our discussion, which we believe address this point.  “Model pre-training offers a particular mechanism through which AI built in data-rich areas might be applied in data-poor regions. Pre-training has grown primarily through NLP, where contextual word embedding models such as Word2Vec, GloVe, and BERT have improved performance of models across a variety of tasks. Pre-trained models are generally trained in one task and subsequently leveraged to solve similar problems. For example, BERT models were built using clinical notes from the publicly available MIMIC-III database for enhanced disease prediction, and have since been used to improve clinical Japanese data representation written in other languages, and integrated with other complementary medical knowledge databases. While development costs can be high (e.g., training GPT-3, a novel large-scale language, cost over $10 million and required more than 300GB of memory), such frameworks negate the limitations of working with smaller datasets through leveraging existing models for recalibration for diverse target populations. Understanding how and why primary models are applicable to different cohorts (i.e., with previously unseen data) also contributes towards refining initial model methodology and proposing robust model alternatives”. | 328 |
| The topic is important. I think a more balanced view, including highlighting the opportunities along with potential fixes as well as the risks, is important to get this message across. | We thank the reviewer for their comments, which the authors unanimously believe improves the quality of our manuscript. We hope we have addressed each of your above-made points suitably. | -- |

| REVIEWER 3 (note: line references refer to the *tracked* manuscript) | | |
| --- | --- | --- |
| Comment | Response | Line |
| It is a very important and interesting manuscript about global disparities through bias in AI. The authors addressed the importance of AI in medical care and through a rigorous and extensive review and application of transfer-learning techniques. They found factors that could account for disparities in building of AI and its application in medical care. | We thank the reviewer for appreciating the importance of our manuscript, and thoughtful suggestions, which we believe improve the quality of our paper. | -- |
| The authors mention in the abstract that databases came mainly from the USA and China. Looking at the results it is noticeable that database, at least the first 10 places, were from high income countries. It is important to introduce a phrase in abstract indicating such observation, because the authors make emphasis, in the interpretation (abstract) that it is important to develop infrastructure for AI in data-poor regions. | We thank the reviewer for drawing this to our attention. Upon inspection of the World Bank classification of *high-income countries* (HICs), China is *not* classified as a HIC – rather, as an *upper-middle income country (UMIC)*.  See: https://datahelpdesk.worldbank.org/  knowledgebase/articles/906519#High_income  However, we agree with the reviewer, and believe this is still worth highlighting: we appreciate the limitations in classification of LIC, LMIC, MIC, UMIC and HIC (being based primarily on GNI per capita), and that China’s population does not accurately reflect the technological advancement/capacity of China.  Indeed, not only are the top 10 databases from HICs (excluding China), but the top 10 author nationalities are *also* from HICs (excluding China).  We have included this in our abstract (line 62): “Here, we found that almost all of the top 10 databases and author nationalities were from high income countries (excluding China), and over half of the databases used to train models came from either the U.S. or China”.  It has also been noted in the opening passage of our discussion (line 309): “We found that the top 10 databases and author nationalities were affiliated with high income countries (excluding China), and over half of the databases used to train models came from either the U.S. or China”.  Please find tracked changes in our manuscript. | 62,  309 |
| In page 16 the authors speak about the over-representation of radiology, the same argument “access to image-data” applies for pathology, which was the other over-represented specialty. | We thank the reviewer for outlining this, which we have noted in our Results:  “Pathology, ophthalmology, neurology, and cardiology comprised four of the following five specialties in both subsets”.  We have also reiterated the potential for image-rich specialties (including pathology) to create collateral benefits for associated specialties (albeit, using Radiology (the commonest specialty) as the example, in our Discussion:  “…our findings also suggest a means through which disparities among clinical specialties could potentially be mitigated: the role that radiology, pathology, ophthalmology, neurology, and cardiology have played in leading the development of clinical AI may yield efficient computational methods that are either applicable or transferrable to other fields”. | 284,  621 |
| The authors do not mention as a limitation on gender analysis, that the same names are used in some countries for women and men. | Thank you for highlighting this to us. We also believe this is an important limitation of our own study (possibly presenting its own bias), and have outlined this on page 17:  “Importantly, the gender inference methods used in this paper, as with any methods outside of direct survey and self-identification, are imperfect and likely to have misgendered a subset of the author population. Additionally, the Genderize.io API was unable to assign a gender to every author name. Further, the API is not inclusive beyond the gender binary (e.g., intersectional gender and they/them pronouns are not considered)”. | 674 |
| Even though AI is very important in Medicine, physicians do not (at least not in many countries) acquire the basic knowledge of AI when they are medical students. Same happens with specialists. Since the manuscript focuses on disparities, it would be convenient to mention the handicap that medical students have in AI knowledge. That explains why a high proportion of the main AI authors are not clinicians. | We thank the reviewer for emphasising that a broader understanding of the role of AI in healthcare (and implications of its use) should be introduced to both clinical and non-clinical researchers (including medical students), as its role/scope in healthcare expands.  We believe this is an important point to note, and have changed our concluding sentence accordingly, to reflect this:  “As the scope of influence of AI in healthcare grows, it is critical for both clinical and non-clinical researchers to understand who may be negatively affected by model bias through underrepresentation and to assess the extent of model utility externally in order to make the vast amount of data being collected and its AI applications meaningful for a broader population”. | 714 |
| Even though I accepted the manuscript I would like the authors to introduce in the abstract that the first ten databases came from high income countries. | We thank the reviewer for their thoughtful, constructive feedback, and the suggestion to note the fact that 9 of the 10 top dataset and author locations were HICs. Please see our response to your second point (in full) above. | 62,  309 |
